# Supplementary material for: Occurrence and Characteristics of Staphylococcus aureus Strains along the Production Chain of Raw Milk Cheeses in Poland
Source: Molecules. 2022 Oct 4;27(19):6569. doi: 10.3390/molecules27196569 (PMC9573400; doi:10.3390/molecules27196569)
Supplement: Supplementary file 1 [file molecules-27-06569-s001.zip › molecules-1916194-supplementary.pdf]

Supplementary materials

Table S1. List of primer sequences used in PCR reactions.

Gene	Primer sequence 5′-3′	Amplicon size (bp)	Annealing temperature	Reference
**Oxacilin resistance gene**				
*mec*A	F: AAAATCGATGGTAAAGGTTGGC<br>R: AGTTCTGGCACTACCGGATTTGC	533	55ºC	[68]
**Tetracycline resistance genes**				
*tet*M	F: GTGGACAAAGGTACAACGAG<br>R: CGGTAAAGTTCGTCACACAC	406	62ºC	[70]
*tet*L	F: TGGTGGAATGATAGCCCATT<br>R: CAGGAATGACAGCACGCTAA	229		
*tet*K	F: TTATGGTGGTTGTAGCTAGAAA<br>R: AAAGGGTTAGAAACTCTTGAAA	348	55ºC	[71]
**Macrolide resistance genes**				
*erm*A	F: TCTAAAAAGCATGTAAAAGAA<br>R: CTTCGATAGTTTATTAATATTAG	645	58ºC	[72]
*erm*B	F: GAAAAGTACTCAACCAAATA<br>R: AGTAACGGTACTTAAATTGTTTA	639		
*erm*C	F: TCAAAACATAATATAGATAAA<br>R: GCTAATATTGTTTAAATCGTCAAT	642		
*msr*A/B	F: TCCAATCATAGCACAAAATC<br>R: AATTCCCTCTATTTGGTGGT	162		
*bla*Z	F: CAAAGATGATATAGTTGCTTATTCTC<br>R: TGCTTGACCACTTTTATCAGC	400	50ºC	[73]
**Aminoglycosides resistance genes**				
aac(6′)-Ie-aph(2′′)-Ia	F: CAGGAATTTATCGAAAATGGTAGAAAAG<br>R: CACAATCGACTAAAGAGTACCAAT	369	58ºC	[74]
aph(3′′)-IIIa	F: GGCTAAAATGAGAATATCACCGG<br>R: CTTTAAAAAATCATACAGCTCGCG	523		
**Biofilm associated genes**				
icaA	F: TCTCTTGCAGGA GCAATCAA<br>R: TCAGGCACTAACATCCAGCA	188	55.5 ºC	[76]
icaB	F: CTGATCAAGAATTTAAATCACAAA<br>R: AAAGTCCCATAAGCCTGTTT	302	56ºC	
icaC	F: TAACTTTAGGCGCATATGTTTT<br>R: TTCCAGTTAGGCTGGTATTG	400	56ºC	[76]
icaD	F: ATGGTCAAGCCCAGACAGAG<br>R: CGTGTTTTCAACATTTAATGCAA	199	55.5 ºC	
bap	F: CCCTATATCGAAGGTGTAGAATTG<br>R: GCTGTTGAAGTTAATACTGTACCTGC	971	60 ºC	[77]
eno	F: ACGTGCAGCAGCTGACT<br>R: CAACAGCATCTTCAGTACCTTC	302	55 ºC	[78]
agrD	F: CATTCCTGTGCGACTTATTAAACG<br>R: CGTGTAATTGTGTAAATTCTTTTGC	307	56ºC	[79]
sarA	F: CGGTACCGTTGATTTGGGTAGTATGC<br>R: TTGCCATGGTTAAAACCTCCC	867	55 ºC	
sigB	F: CGGATCCGGTGTGACAATCAGTATGAC<br>R: CGGAATTCGCGACATTTATGTGGATACAC	937	55 ºC	
**Enterotoxigenicity genes**				
sea	sea 1: GAAAAAAGTCTGAATTGCAGGGAACA<br>sea 2: CAAATAAATCGTAATTAACCGAAGGTTC	560	55 ºC	[81]
seh	seh 1: CAATCACATCATATGCGAAAGCAG<br>seh 2: CATCTACCCAAACATTAGCACC	376		

sec	sec 1: CTTGTATGTATGGAGGAATAACAAAACATG<br>sec2 :CATATCATACCAAAAAGTATTGCCGT	275	
tst-1	tst1: TTCACTATTTGTAAAAGTGTCAGACCCACT<br>tst 2: TACTAATGAATTTTTTTATCGTAAGCCCTT	180	
sed	sed 1:GAATTAAGTAGTACCGCGCTAAATAATATG<br>sed 2: GCTGTATTTTTCCTCCGAGAGT	492	
etd	etd1: CAAACTATCATGTATCAAGGATGG<br>etd2: CCAGAATTTCCCGACTCAG	358	[61]
eta	eta 1: ACTGTAGGAGCTAGTGCATTTGT<br>eta2: TGGATACTTTTGTCTATCTTTTTCATCAAC	190	
selk	sek1: ATGCCAGCGCTCAAGGC<br>sek2: AGATTCATTTGAAAATTGTAGTTGATTAGCT<br>sek3: TGCCAGCGCTCAAGGTG	134	[82]
see	see1: CAAAGAAATGCTTTAAGCAATCTTAGGC<br>see2: CACCTTACCGCCAAAGCTG	482	
seb	seb1: ATTCTATTAAGGACACTAAGTTAGGGA<br>seb2: ATCCCGTTTCATAAGGCGAGT	404	[81]
selm	sem-1: CTATTAATCTTTGGGTTAATGGAGAAC<br>sem-2 : TTCAGTTTCGACAGTTTTGTTGTCAT	326	
sell	sel-1 : GCGATGTAGGTCCAGGAAAC<br>sel-2 : CATATATAGTACGAGAGTTAGAACCATA	234	
selo	seo-1 : AGTTTGTGTAAGAAGTCAAGTGTAGA<br>seo-2: ATCTTTAAATTCAGCAGATATTCCATCTAAC	474	
seln	sen-1: CGTGGCAATTAGACGAGTC<br>sen-2 : GATTGATYTTGATGATTATKAG	180	
seg	seg-1: TCTCCACCTGTTGAAGG<br>seg-2: AAGTGATTGTCTATTGTCG	323	
selq	seq-1: ACCTGAAAAGCTTCAAGGA<br>seq-2: CGCCAACGTAATTCCAC	204	
selj	sej-1: TCAGAACTGTTGTTCCGCTAG<br>sej-2: GAATTTTACCAYCAAAGGTAC	138	[82]
sei	sei-1: CTT GAA TTT TCA ACM GGT AC<br>sei-2: AGG CAG TCC ATC TCC TG	461	
ser	ser-1: AGCGGTAATAGCAGAAAATG<br>ser-2: TCTTGTACCGTAACCGTTTT	363	
selu	seu-1: AATGGCTCTAAAATTGATGG<br>seu-2 : ATTTGATTTCCATCATGCTC	215	
selp	sep-1: GAATTGCAGGGAACTGCT<br>sep-2: GGCGGTGTCTTTTGAAC	182	
